# Nanocarrier-Based Eco-Friendly RNA Pesticides for Sustainable Management of Plant Pathogens and Pests

**DOI:** 10.3390/nano14231874

**Published:** 2024-11-22

**Authors:** Heng Qiao, Jingyi Chen, Min Dong, Jie Shen, Shuo Yan

**Affiliations:** Department of Plant Biosecurity, College of Plant Protection, China Agricultural University, Beijing 100193, China; qiaoheng.tao@cau.edu.cn (H.Q.); chenjingyi@cau.edu.cn (J.C.); dongmin@cau.edu.cn (M.D.); shenjie@cau.edu.cn (J.S.)

**Keywords:** dsRNA delivery system, nanoparticles, pathogens and pests management, sustainable agriculture, RNA pesticide

## Abstract

The production of healthy agricultural products has increased the demand for innovative and sustainable plant protection technologies. RNA interference (RNAi), described as post-transcriptional gene silencing, offers great opportunities for developing RNA pesticides for sustainable disease and pest control. Compared with traditional synthesized pesticides, RNA pesticides possess many advantages, such as strong targeting, good environmental compatibility, and an easy development process. In this review, we systematically introduce the development of RNAi technology, highlight the advantages of RNA pesticides, and illustrate the challenges faced in developing high-efficiency RNA pesticides and the benefits of nanocarriers. Furthermore, we introduce the process and mechanism of nanocarrier-mediated RNAi technology, summarize the applications of RNA pesticides in controlling plant pathogens and pests, and finally outline the current challenges and future prospects. The current review provides theoretical guidance for the in-depth research and diversified development of RNA pesticides, which can promote the development and practice of nanocarrier-mediated RNAi.

## 1. Introduction

The production and safety of food have always been the primary problems for human survival and social development. In recent years, crop diseases and pests have caused substantial economic losses for agricultural production throughout the world. To address the escalating demands of a continuously growing global population, traditional synthesized pesticides have been extensively utilized to mitigate agricultural losses due to plant diseases and pests, thereby enhancing the quality and quantity of agricultural production. However, their unscientific application poses a grave threat to both environmental and human health [1,2]. Therefore, the development of efficient and eco-friendly plant protection technology has become a major global strategic demand.

As a highly conserved, sequence-specific method for inhibiting gene expression, RNA interference (RNAi) was first identified in the nematode *Caenorhabditis elegans* by Andrew Z. Fire and Craig C. Mello, who received the 2006 Nobel Prize in Physiology or Medicine [3]. Since its discovery, RNAi has swiftly emerged as a potent reverse genetic tool for exploring gene function, regulation, and interaction at both the cellular and organismal levels, while also demonstrating immense potential in controlling plant pathogens and pests [4,5,6,7,8]. RNA pesticides are mainly based on the RNAi principle of inhibiting the expression of important functional genes in harmful organisms, resulting in their developmental retardation or death [9,10,11] and are regarded as “the third revolution in the history of pesticides” due to their extensive advantages such as precision, high efficiency, being pollution-free, etc. [12]. However, the RNAi effect is moderate or temporary in many recalcitrant pests and pathogenic microorganisms, and the major challenges include double-stranded RNA (dsRNA) instability in the environment and organisms, dsRNA accumulation in lysosomes, and low delivery efficiency of dsRNA across various biological physical barriers (i.e., the insect body wall and microbial cell wall) [6,13,14,15,16,17]. Therefore, it is necessary to develop an efficient dsRNA delivery system to increase the stability and delivery efficiency of dsRNA.

In the past decade, nanotechnology has been widely used in various fields such as medicine, electronics, aerospace, life sciences, and agriculture [18,19]. Nanomaterials are regarded as the most promising materials in the 21st century due to their excellent physical and chemical properties, and they have been extensively applied to construct nano-delivery platforms, thus becoming a research hotspot in the fields of medicine and modern agriculture [20,21]. Currently, nanocarrier-mediated RNAi has been reported as a novel method for delivering dsRNA to improve RNAi efficiency [22,23,24,25]. Nanocarriers can assemble with nucleic acids through electrostatic interaction, hydrogen bonding, and Van der Waals forces. In the process of dsRNA delivery, nanocarriers can protect dsRNA from degradation by nucleases in organisms, increase dsRNA uptake via activating clathrin-mediated endocytosis, and aid dsRNA to achieve early endosomal escape, thereby improving RNAi efficiency [26]. In this review, we systematically introduced the development of RNAi technology, summarized the advantages of RNA pesticides, expounded the advances, process, and mechanism of nanocarrier-mediated RNAi, discussed the application of RNA pesticides in controlling pests and diseases, and finally put forward the current challenges and future prospects.

## 2. RNAi Technology for Gene Function Analysis and Pesticide Development

RNAi is a widespread post-transcriptional gene silencing phenomenon in eukaryotes, triggered by the efficient and specific degradation of homologous mRNA by double-stranded RNA (dsRNA) [27]. To date, three major RNAi pathways have been identified in organisms, such as the short/small interfering RNA (siRNA) pathway, microRNA pathway, and Piwi-interacting RNA pathway [6]. Among them, siRNA is a kind of double-stranded RNA with a length of 19–21 bp, and it is the most widely studied pathway. When exogenous dsRNA enters the cell interior, it is cleaved by Dicer2 into siRNAs of 19–21 bp in length [28,29]. Subsequently, siRNA binds to the RNA-induced silencing complex (RISC) assembled by Ago2 and other RISC-associated proteins (RP) and unwinds into single-stranded RNA. The sense strand is degraded, while the antisense strand guides the cleavage of the complementary target mRNA, thereby achieving gene silencing [6,30].

For gene function analysis in insects, RNAi was initially applied in the model insect *Drosophila melanogaster*. Kennerdell and Carthew [31] initially conducted RNAi research in *Drosophila*, and successfully confirmed that two genes, *frizzled* and *frizzled2*, belong to the wingless signaling pathway. So far, RNAi, as a powerful research tool, has helped researchers perform large-scale identification of functional genes in many economically important insects. RNAi technology is not only a powerful research tool but also a novel and safe strategy for plant protection [4,32]. RNAi can be applied to inhibit the expression of important genes for pest growth, causing developmental disorders or death [33,34]. Thus, it is specific, safe, and easy to operate, making it suitable to be applied to the development of RNA pesticides [6,35]. In 2007, Monsanto, a company in the United States, successfully developed transgenic maize that expresses *V-ATPase A* dsRNA, which can be applied to control the western corn rootworm (WCR) *Diabrotica virgifera virgifera* [4]. Subsequently, this transgenic maize was approved by the Food and Drug Administration (FDA) for commercial cultivation in 2017. For the development of a sprayable dsRNA product, Greenlight Biosciences announced a dsRNA product named Ledprona for controlling the Colorado potato beetle *Leptinotarsa decemlineata* [36], which was approved by the United States Environmental Protection Agency (EPA) in December 2023, making it the world’s first sprayable RNA biopesticide for commercial application.

## 3. Advantages of RNA Pesticide Compared with Traditional Pesticides

At present, the long-term use of chemical pesticides results in pesticide resistance and residues, leading to environmental pollution, higher control costs, and serious biosecurity problems [2]. RNA pesticides, as a new pest control strategy, have many features and advantages compared with traditional pesticides [7,37]. RNAi is highly specific, and its sequence-dependent mode of action has also attracted great interest in crop protection [27,28]. RNAi triggers, such as artificial microRNAs, hairpin-structured RNAs, and dsRNAs, can be specifically designed to selectively target the specific genes in harmful organisms [38]. Thus, it is possible to achieve specific control of a target species or group of species without harming non-target species. Related studies have proved that the use of RNA pesticides can effectively control tomato leaf miner (*Tuta absoluta*) and cotton bollworm (*Helicoverpa armigera*) without affecting non-target organisms [39,40]. The RNAi strategy can also be applied to reduce honeybee parasites such as *Varroa mites* and internal microsporidian parasites without harmful effects on bees [41,42,43]. In addition, it has been demonstrated that dsRNA has a short duration in the environment, with analyses of soil and plants treated with dsRNA showing that dsRNA breaks down rapidly, within 2–3 d, which means that there are fewer concerns about the unintended contamination of water supplies, soils, and other environmental factors [44,45]. In addition, since all organisms have evolved to break down dsRNA and apply the nucleic acids as cellular nutrients, this technology would be safer than traditional pesticides [46,47,48].

The simple process and low costs for developing RNA drugs/pesticides are great advantages compared to traditional drugs/pesticides. In the medical field, the development time of novel coronavirus mRNA vaccines has been shortened to 11 months compared to several years for traditional vaccines [49]. The same is true for RNA pesticides, which have a much shorter development cycle compared to traditional pesticides. Statistically, the successful marketing of a new pesticide species requires an average of 160,000 compounds to be screened, at a cost of about 300 million dollars and a time frame of 12 years [50]. When the production technology and formulation technology for dsRNA are solved, it will only be necessary to screen new targets to develop spray-type RNA pesticides. In addition, RNA pesticides can also be designed and tested faster (in about 2–3 years) than transgenic crops, which can take 10 to 20 years and cost hundreds of millions of dollars [51].

Transgenic technologies have been successfully applied to develop transgenic crops with improved plant disease and pest resistance via RNAi strategies [10,47]. Recently, several studies have highlighted the promising potential of this strategy in plant protection. Researchers have demonstrated that the expression of dsRNA targeting the effector *Avra10* of *Blumeria graminis* in both barley (*Hordeum vulgare*) and wheat (*Triticum aestivum*) can inhibit *Avra10* expression, affecting the growth of the powdery mildew fungus and consequently diminishing its pathogenicity [52]. The first genetically modified (GM) maize crop (MON87411), expressing dsRNA against the WCR, has received approval for cultivation in more than 15 countries [10]. However, the transformative RNAi-based approach faces expensive capital requirements and the public acceptance of GM crops. Non-transformative RNA pesticides can be used in the same convenient and diversified ways as synthesized pesticides, such as in sprays, root/seed soaking, trunk injection, and petiole absorption, compared with GM crops [34,53,54]. For instance, researchers have reported that bacterially expressed ds*HvSnf7*, applied to detached plant leaves, causes 98% mortality in *Henosepilachna vigintioctopunctata* [55]. In crop disease control, spray-induced gene silencing (SIGS) technology has been applied to a variety of crops, such as rice (*Oryza sativa*) and tomato (*Solanum lycopersicum*) [16,56,57]. In summary, these studies have highlighted the potential of utilizing non-transformative RNA pesticides to control plant pathogens and pests.

## 4. Bottlenecks for Developing High-Efficiency RNA Pesticides

### 4.1. Instability of dsRNA

**Figure 1:**
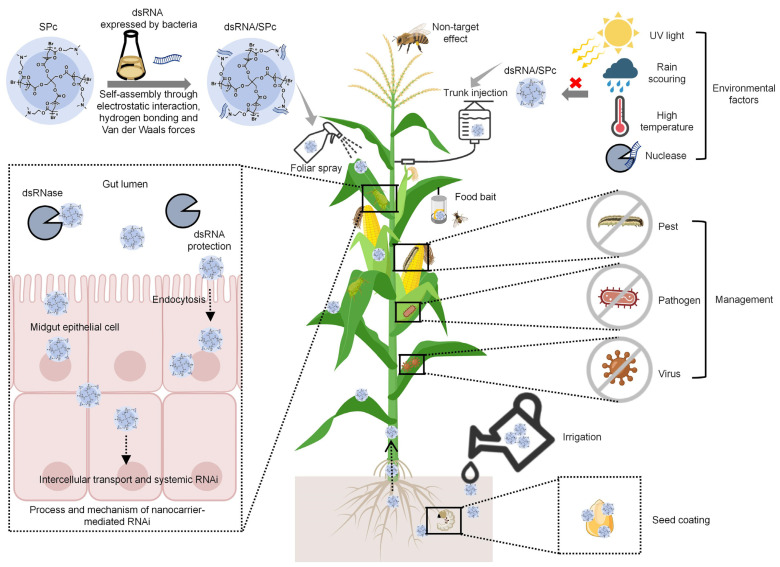
Application of star polymer (SPc) nanocarrier to improve RNAi efficiency for plant pathogen and pest management. The figure was created using BioRender.com and PowerPoint 2019 software (Microsoft, Redmond, WA, USA).

dsRNAs are environmentally unstable and are susceptible to degradation by UV light, rain scouring, high temperature, and nucleases (Figure 1) [34,58]. The dsRNases inside insects or pathogenic microorganisms can affect the stability of dsRNA, which seriously affects the interference efficiency of RNA pesticides in harmful organisms [59]. For instance, the expression suppression of *dsRNase* genes in *L. decemlineata*, *Schistocerca gregaria*, *Manduca sexta*, and *Apolygus lucorum* by RNAi can significantly reduce the degradation of dsRNAs and enhance the stability and persistence of dsRNAs in insects [60,61,62,63]. In addition, the pH environments in insects can potentially affect RNAi efficiency. The physiological pH values of insect hemolymph are generally between 6.4 and 7.5 but vary greatly in the digestive guts [53]. On the one hand, RNA is highly prone to hydrolysis in environments of pH > 6.0 or <3.0 [54]. On the other hand, dsRNases exert higher enzyme activity under alkaline conditions to promote dsRNA degradation [55,56]. For instance, *LmdsRNase2* is highly expressed in the midgut of *Locusta migratoria* and rapidly degrades dsRNA under alkaline extracellular conditions [15,64]. Many lepidopteran insects that are less sensitive to RNAi also have strong nuclease activity in the alkaline gut environment [65,66].

### 4.2. Obstacles for Delivering dsRNA

There are two obstacles for the delivery of dsRNA. One is the way to deliver dsRNA in vitro. Various methods have been tested for dsRNA delivery in insect species, including microinjection, oral feeding, and so on [67,68,69,70]. The advantage of microinjection is that it can deliver a precise amount of dsRNA into target tissues, and it is suitable for the functional identification of genes, not for field application [71]. Oral feeding is obviously more convenient and suitable for application in the field, but dsRNA is affected by both the external environment and the presence of dsRNases [14,72]. The other obstacle is the absorption, transport, and escape of dsRNA in vivo. Studies have shown that exogenous dsRNA enters cells mainly through systemic RNAi deficiency (Sid) protein-mediated uptake and clathrin-dependent endocytosis. Winston et al. [73] first found that *Sid-1* was involved in the uptake of dsRNA in *C. elegans*. Subsequently, *Sid-1* homologs have been identified in insects, but the *Sid-1*-like genes in insects do not mediate the uptake of dsRNA [74]. Since Ulvila et al. [75,76,77] first discovered that dsRNA enters *D. melanogaster* S2 cells via the clathrin-dependent endocytosis pathway, most publications have supported this mechanism. Bafilomycin-A (Baf A) can inhibit the efflux of protons across the plasma membrane and has been widely applied to block clathrin-mediated endocytosis [78]. The application of Baf A in *S. frugiperda* results in the failure of cellular uptake of dsRNA and the loss of biological function of ds*ATP-d* [26]. When exogenous dsRNAs enter the cell, they rapidly enter the early endosomes, translocate into the late endosomes with the participation of Rab and other proteins, and then fuse with lysosomes to be degraded [29,71]. Therefore, RNAi efficiency is also contingent upon dsRNA’s successful escape from both early and late endosomes.

## 5. Process and Mechanism of Nanocarrier-Mediated RNAi

**Figure 2:**
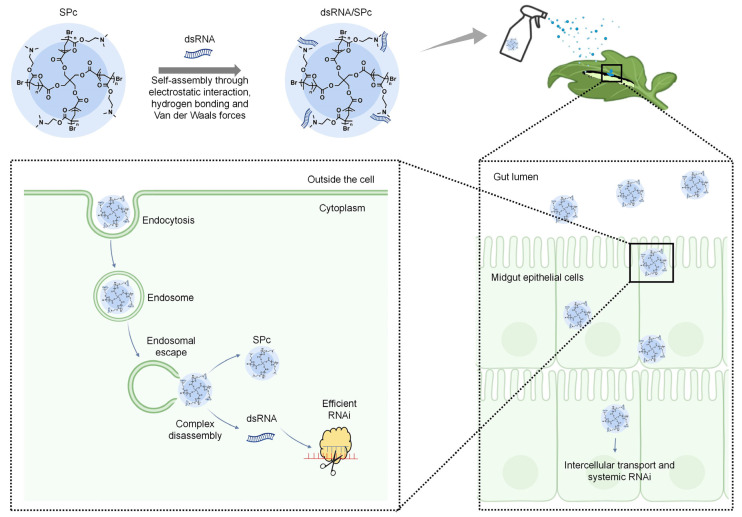
Schematic representation of SPc-mediated dsRNA delivery system. The figure was created using BioRender.com and PowerPoint 2019 software (Microsoft, Redmond, WA, USA).

The low stability and delivery efficiency of dsRNA have been restricting the development of high-efficiency RNA pesticides. Recently, studies have demonstrated that the stability of dsRNA can be enhanced through the strategy of nanocarrier-loaded dsRNA [54,71]. The process and mechanism of nanocarrier-mediated dsRNA delivery can be summarized into four main steps: nucleic acid binding or encapsulation, cellular uptake, endosomal escape, and the release of nucleic acids or degradation of nanocomplexes (Figure 2).

In most cases, positively charged nanocarriers can self-assemble with negatively charged dsRNA to form dsRNA/nanocarrier complexes via electrostatic interaction [79,80]. Additionally, hydrogen bonding and Van der Waals forces contribute significantly to the self-assembly process of complexes [26]. For instance, chitosan possesses large numbers of positively charged amino groups under slightly acidic conditions, which can interact electrostatically with negatively charged dsRNA to form dsRNA/chitosan complexes [81,82]. Another nanocarrier that possesses tertiary amines, such as SPc, can also be loaded with dsRNA by electrostatic interaction. Additionally, hydrogen bonding and Van der Waals forces also play significant roles in the self-assembly of dsRNA/SPc complexes [26].

The dsRNA/nanocarrier complexes are typically positively charged, facilitating interactions with the negatively charged cell membrane [83]. Upon interaction with the cell membrane, dsRNA/nanocarrier complexes enter the cell mainly through receptor-mediated endocytosis [84,85,86]. For instance, SPc can activate clathrin-mediated endocytosis by up-regulating some key genes such as *Chc*, *AP2S1*, and *Arf1*. The suppression of endocytosis can hinder the cellular uptake of SPc-delivered dsRNA in vitro, and the subsequent RNAi effect also disappears in vivo [26]. Additionally, SPc can also activate the endocytosis pathway of potato plants to amplify the defense responses induced by the chitosan elicitor against potato late blight [87].

After the cellular uptake, dsRNA/nanocarrier complexes are typically coated by membrane-bound vesicles called endosomes. If complexes fail to escape from the endosome, they will ultimately be degraded within the lysosome along with the endosome [88,89]. The mechanism of endosomal escape remains controversial, with the “proton sponge” effect being one of its widely accepted hypotheses. For instance, many cationic polymers exhibit a robust buffering capacity within a pH range of 5 to 7, and this acidic environment can result in the protonation of their amine groups, thereby causing a water influx that leads to endosome lysis [84,90]. Compared to naked dsRNA, the dsRNA/SPc complex rapidly disperses from early nuclear endosomes into the cytoplasm, and there is nearly no accumulation of SPc-delivered dsRNA in the late endosomes, suggesting that SPc promotes the endosomal escape of dsRNA [26]. Furthermore, cationic lipid nanoparticles have a unique endosomal escape mechanism that destabilizes endosomes by recognizing and binding to phospholipids on the endosomal membranes, thus accomplishing endosomal escape [91,92].

After the endosomal escape, dsRNA/siRNA must be released from the nanocarrier to activate the RNAi pathway and exert its biological effects. However, there are few studies on the mechanism of dsRNA/siRNA release from nanocarriers, which primarily involves two release mechanisms. One mechanism is a slow competitive displacement process, which is the process of replacing and releasing dsRNA/siRNA by various highly charged polyanions, such as heparin, chondroitin sulfate, and other analogues with high affinity for nanocarriers [93,94,95]. Another mechanism is based on the response of nanocarriers to intracellular stimuli. The physical or chemical properties of nanocarriers can be altered in response to stimulation by intracellular stimuli such as acidic pH and cytosolic reducers. For instance, poly (β-amino ester) nanocarriers respond to environmental pH changes, while disulfide-containing (S-S) nanocarriers are stimulated by intracellular glutathione redox reactions, and these changes aid the release of dsRNA/siRNA [86,96,97].

## 6. Potential Application of Nanocarrier-Based RNA Pesticides

### 6.1. Successful Cases of Nanocarrier-Mediated RNAi

In recent years, various nanocarriers have been designed for the delivery of dsRNA to overcome the delivery barriers of dsRNA, and they have been successfully applied in pest control (Table 1). In insect RNAi, chitosan nanocarrier-loaded dsRNA was first used for silencing *chitin synthase* genes in *Anopheles gambiae*, resulting in the *AgCHS1* transcript level and chitin content being reduced by 62.8% and 33.8%, respectively [81]. Furthermore, the chitosan coupled with cross-linkers such as sodium tripolyphosphate, polyethylene glycol, and polyethyleneimine can enhance the transfection efficiency of dsRNA/siRNA and protect dsRNA/siRNA from degradation [98,99]. Carbon quantum dots (CQD) and liposomal lipofectamine 2000 nanocarriers can significantly improve RNAi efficiency and mortality of *Chilo suppressalis* [100]. He et al. [101] and Zheng et al. [23] successfully constructed a series of cationic core-shell fluorescent nanocarriers based on perylene diimide, which can significantly down-regulate the expression of target genes in *Agrotis ypsilon* and *Aphis glycines*, inhibiting their growth and development via various delivery methods such as injection, feeding, and topical application. An SPc-based transdermal dsRNA delivery system was successfully constructed and applied to a variety of lepidopteran and hemipteran pests, such as *A. ypsilon*, *S. frugiperda*, *A. glycines*, *Myzus persicae*, etc. [24,102,103,104]. Additionally, nanocarriers can also improve RNAi efficiency in plant pathogenic microorganisms (Table 2). For instance, dsRNA-CDs display excellent control effects on *Phytophthora infestans*, *P. sojae*, and both the wild-type and fungicide-resistant *P. capsici.* [25]. Artificial nanovesicles (AVs) have been synthesized by using three different cationic lipid formulations, which can improve dsRNA stability, leading to prolonged RNAi-mediated protection against the fungal pathogen (*Botrytis cinerea*) in both pre- and post-harvest plants [105].

### 6.2. Application Method of Nanocarrier-Based RNA Pesticides

For nanoparticle-based RNA pesticide applications, foliar spray, irrigation, and trunk injection would be good choices to improve bioactivity (Figure 1). Crops can be sprayed directly with RNA pesticides that target plant pathogens or pests. GreenLight Biosciences has developed sprayable RNA pesticides for controlling *V. mites*, *L. decemlineata*, powdery mildew, gray mold, and so on [59]. Among them, Ledprona is a sprayed dsRNA product for controlling *L. decemlineata* [36], which has been approved by the EPA for commercialization. Nanocarriers can also promote the delivery of dsRNA through root application in *Arabidopsis* and maize, which is conducive to the development of irrigation and trunk injection [133]. For example, SPc can deliver nucleic acids into plants though root application, which leads to the gene silencing and wing aberration of green peach aphids feeding on plants [120]. In addition, food bait and seed coating are also potential application methods for nanocarrier-based RNA pesticides.

## 7. Current Challenges and Future Perspective

### 7.1. Control Efficacy of RNA Pesticides

RNAi efficiency varies greatly among different insect species, being high in some coleopterans and orthopterans, such as *Tribolium castaneum*, *L. decemlineata*, *L. migratoria*, and *S. gregaria* [134,135,136], but low in lepidopterans, hemipterans, and others [72,137]. Even if different insect species have the same genes or transcripts, RNAi efficiency can vary greatly among them [138]. Therefore, it is necessary to continuously screen for specific RNAi target genes that have high lethal effects. In addition, the dsRNAs targeting multiple genes have shown potential for synergistic effects. For instance, larvae fed with ds*IAP* and ds*COP* sequentially show a higher mortality (55%) than those fed with only ds*IAP* (33%) or ds*COP* (24%) in *Agrilus planipennis* [139]. The simultaneous ingestion of both dsRNAs at low concentrations (1 μg/μL) results in up to 90% mortality, whereas a single dsRNA treatment shows similar mortality but at much higher concentrations (10 μg/μL) [140]. Nanocarriers can be also loaded with dsRNAs of multiple genes for further enhanced synergistic effects. In *T. castaneum*, larvae fed with both dsRNAs complexed with BACPs have 20% and 30% higher mortality rates than those fed with ds*BiP*/BACPs or ds*Armet*/BACPs alone, respectively [124]. Ma et al. [104] sprayed an SPc-loaded dsRNA formulation, co-targeting *V-type proton ATPase subunits d* (*ATP-d*) and *G* (*ATP-G*) of *M. persicae*, with a high control efficacy of 61% at 3 d, but its bioactivity and persistence were not as good as those of synthetic/botanical pesticides.

To improve the control effect of RNA pesticides, our team has constructed a novel multicomponent nano-pesticide with co-delivery of dsRNA and pesticides. This co-delivery can achieve high bioactivity and long persistence for sustainable disease and pest management. For instance, Li et al. [141] constructed a novel multicomponent nano-pesticide by using SPc for co-delivering *hemocytin* dsRNA and botanical pesticide matrine, which improves the persistence of dsRNA and overcomes the slow action of matrine. Yan et al. [142] developed a multicomponent nano-pesticide (pesticide/SPc/ds*Nrf2* complex) using a bacterial expression system and nano-delivery system. It had a good control effect on *S. frugiperda*, and its control efficacy remained at 94.91% at 7 d after application, while it was only 62.69% for pesticide alone. In addition, the development of multicomponent nano-bioprotectants is also an eco-friendly strategy to manage plant pathogens. Herein, a self-assembled multicomponent nano-bioprotectant for potato late blight is designed based on dsRNA and a plant elicitor, and it displays a good protective effect (68%) compared with the widely used mancozeb fungicide (53%) [143]. Moreover, in addition to the management of agricultural diseases and pests, RNA pesticides can be also used in combination with chemical herbicides to control weeds, which is regarded as a new strategy for weed control in the future.

### 7.2. Risk Assessment of RNA Pesticides

The long-term use of traditional chemical pesticides can lead to pest resistance, and plant pathogens and pests can also develop resistance to RNA pesticides. Insects may evolve resistance to RNA insecticides through mismatches between dsRNA and target mRNA sequences caused by genetic mutations or polymorphisms [144]. Insects can also become resistant to RNA pesticides by preventing cells from taking up dsRNA. For example, a *DvSnf7* dsRNA-resistant WCR population was screened and established by a field experiment, and the Cy3-labeled *DvSnf7* dsRNA was observed in midgut cells of the dsRNA-sensitive population, but not in the resistant population, indicating that the dsRNA uptake by midgut cells is impaired in resistant WCR [145]. Liao et al. [146] screened resistant willow leaf beetle (*Plagiodera versicolora*) in the laboratory and found that the development of resistance may be related to the impaired uptake of dsRNA by midgut cells. In addition, the down-regulation or mutation of genes in RNAi machinery may also be a potential mechanism for the development of dsRNA resistance. Yoon et al. [69] identified the dsRNA binding protein StaufenC as a major player for processing dsRNA into siRNA, but the resistant cells show lower expression of StaufenC, which may be a potential target for overcoming dsRNA resistance.

In addition to the resistance problem, another problem associated with the application of RNA pesticides is the unintended effects on non-targets. RNA insecticides have a relatively narrow insecticidal spectrum, and species taxonomically related to the target pest are more likely to be susceptible [147]. Therefore, to avoid the potential risks of RNAi products, researchers need to exclude the risks that dsRNAs may pose to non-target organisms in a sequence-specific manner when designing RNA pesticides. In addition, although the application of nanocarriers may improve the persistence of RNA pesticides, nanocarriers may introduce new environmental and human health hazards, especially the biotoxicity of nanocarriers. For example, SPc can damage the cell membrane and nucleus of intestinal tissue at very high concentrations but does not show toxicity at working concentrations [148]. Consequently, strict application guidelines must be followed when it is used at a large scale. Additionally, the concentration of dsRNA should be also noted. High applied concentrations of dsRNA may saturate the core mechanism of RNAi and activate the immune system, which may have harmful effects on organisms [149].

### 7.3. Biosynthesis of dsRNA

To reduce the production cost of dsRNA for field applications, large-scale dsRNA synthesis using microbial fermentation is considered one of the most promising methods. Recently, dsRNA has been expressed and synthesized in a variety of microorganisms, including *Escherichia coli*, *Bacillus subtilis*, *Saccharomyces cerevisiae*, etc. [150,151,152]. The L4440-HT115 system is the most widely used dsRNA expression system and has been successfully applied in the RNAi of *Mythimna separate*, *L. decemlineata*, *Bactrocera dorsalis*, etc. [153,154,155]. However, its synthesis efficiency and yield are still not enough to satisfy large-scale field application. Currently, researchers constructed an innovative pET28-BL21 (DE3) RNase III- system for the efficient expression of large batches of dsRNA, boasting a dsRNA expression efficiency that is three times higher than that of the widely utilized L4440-HT115 (DE3) system [156]. In addition, the cost of dsRNA production has been reduced from USD12,000/g in 2008 to USD1/g in 2021 through the continuous endeavors of many biological companies [34,54,157]. Among them, GreenLight Biosciences has further reduced the production cost of dsRNA to USD0.5/g for field applications [54,158]. Thus, it appears that the current cost of dsRNA production is fully compatible with the commercialization of RNA pesticides.

## Figures and Tables

**Table 1:** Applications of nanocarrier-mediated RNA pesticides for pest management.

Nanoparticle	Insect	Target Gene	Delivery Method	Reference
Chitosan	*Chilo suppressalis*	*CHSA*, *CHSB*, *G3PDH*	Oral feeding	[100]
	*Spodoptera frugiperda*	*IAP*	Oral feeding	[106]
	*Apolygus lucorum*	*GRK2*	Oral feeding	[82]
	*Bemisia tabaci*	*ECR*	Oral feeding	[107]
	*Tetranychus cinnabarinus*	*TcCHIT10*, *Tcβ-COP*, *TcCHC*	Oral feeding	[108]
	*Aedes aegypti*	*SNF7*	Oral feeding	[109]
		*Sema1a*	Oral feeding	[110]
		*DOPAL synthase*	Oral feeding	[111]
		*Vestigial gene*	Oral feeding	[112]
	*Anopheles gambiae*	*Chitin synthase 1*	Oral feeding	[81]
		*Chitin synthase 2*	Oral feeding	[110]
		*Cadherin1*, *Cadherin2*	Oral feeding	[113]
PEG–Chitosan	*Nilaparvata lugens*	*NlCHSA*	Topical application	[114]
Chitosan–sodium tripolyphosphate	*Aedes aegypti*	*IAP*	Oral feeding	[98]
	*Helicoverpa armigera*	*JHAMT*, *ACHE*	Oral feeding	[40]
		*Lipase*, *chitinase*	Oral feeding	[115]
Liposom	*Drosophila melanogaster*	*V-ATPaseE*	Soaking and oral feeding	[38]
	*Drosophila suzukii*	*Vha26*	Oral feeding	[116]
	*Euschistus heros*	*V-ATPaseA0*, *Muscle actin*	Oral feeding	[117]
	*Chilo suppressalis*	*CHSA*, *CHSB*, *G3PDH*	Oral feeding	[100]
	*Spodoptera frugiperda*	*IAP*	Oral feeding	[118]
Perylenediimide-cored cationic dendrimers	*Ostrinia furnacalis*	*Serpin-3*	Oral feeding	[22]
		*Chitinase-like*, *CHT10*	Microinjection and oralfeeding	[101]
	*Aphis glycines*	*Hemocytin*	Topical application	[23]
Star polycation	*Agrotis ypsilon*	*V-ATPase*	Microinjection and oralfeeding	[24]
	*Spodoptera frugiperda*	*V-ATPaseD*, *chitin synthase1*	Soaking, topical application, and oral feeding	[102]
		*ECR*	Spraying	[119]
	*Aphis glycines*	*Treh*, *V-ATPaseD*, *V-ATPaseE*, *chitin synthase1*	Topical application and spraying	[103]
	*Myzus persicae*	*vestigial*, *ultrabithorax*	Topical application	[120]
	*Aphis gossypii*	*AgCHS2*, *AgHK2*	Spraying	[121]
	*Blattella germanica*	*BgCHS1*, *BgCHS2*	Oral feeding	[122]
Block copolymer	*Locust migratoria*	*LmCHS1*, *LmCHS2*	Oral feeding	[123]
Branched amphiphilic Peptide capsules	*Acyrthosiphon pisum*	*Armet*, *BiP*	Oral feeding	[124]
	*Tribolium castaneum*	*Armet*, *BiP*	Oral feeding	[124]
Carbon quantum dot	*Chilo suppressalis*	*CHSA*, *CHSB*, *G3PDH*	Oral feeding	[100]
Metal organic framework	*Nilaparvata lugens*	*NlCYP303A1*	Oral feeding	[125]
Graphene oxide	*Drosophila suzukii*	*vATPase*	Oral feeding	[126]

**Table 2:** Applications of nanocarrier-mediated RNA pesticides for plant pathogen management.

Nanoparticle	Host	Pathogen	Target Gene	Reference
Star polycation	rice	*Rhizoctonia solani*	*RsAGO1*, *RsAGO2*	[127]
Artificial nanovesicles	tomato, grape	*Botrytis cinerea*	*Dicer-like 1*, *Dicer-like 2*	[105]
Layered double hydroxide	cowpea	Common mosaic virus	*CMV2b*	[128]
		Bean common Mosaic virus	*Coat protein*	[129]
	grape, cherry	*Botrytis cinerea*	*erg13*, *erg11*, *erg1*	[130]
	maize	*Rhizoctonia solani*	*RsCRZ1*	[131]
	tomato	*Botrytis cinerea*	*BcDCL1/2, BcVDS*	[132]
Carbon dot	*Nicotiana benthamiana*, chili	*Phytophthora infestans*, *Phytophthora sojae*, *Phytophthora capsici*	*CesA3*, *OSBP1*	[25]
